# Vain Hopes

**Published:** 1870-04

**Authors:** 


### VAIN HOPES.

CRUSHED hopes often kill and sometimes in a moment; generally they sadden for a time, and then they are obliterated from the memory, by the occupations and excitements of life; but the repetitions become so frequent in the course of years, that at threescore hope is almost exhausted, men habitually shake their heads at all plans and schemes that promise anything beyond the most ordinary results, while the loving parent heaves a heavy sigh when the thought sweeps across the heart that the child of his affections has to pass through the sad experience which he has done, and for a moment almost wishes that he had been laid in an early grave. There is only one antidote to life's disappointments, and that is to accustom the mind to look forward to the end, to the great fact of one anticipation that can never fail of realization, and which is so stupendous in its nature, so resplendent, that all other desires and expectations pale away in utter insignificance; and that is a well-grounded hope that when the mortal body is laid in the grave, the immortal part, the man himself, shall pass into an immortal existence, to dwell forever in the bosom of the great Father of us all.

But while it is true that blasted hopes are sufficient at times to destroy life, and at others undermine the health and render the unfortunate individual unfit for the discharge of life's duties, unless there is force of character enough to rouse the soul from its lethargy, and bravely engage in the battle of life again, it is also true that the sudden realization of a long and anxiously cherished desire and expectation is sufficient to destroy life in a moment. Cases are on record where persons have dropped down dead on being informed of drawing a large prize in a lottery. Other cases there are of persons being thrown into uncontrollable laughter at hearing of some glad event; a laughter, natural at first, but soon becoming nervous and convulsive, resulting in death.

There is an institution in England for furnishing a comfortable home for a certain class of old men, — a delightful home for life ; only a certain number can be accommodated ; when vacancies are made by death or otherwise, it usually happens that there are a dozen times as many applicants in waiting as there are places for. At a recent election two old men dropped dead on the announcement that they were entitled to vacancies. But in less serious cases of blasted hopes on the part of persons who write for fame or money, what millions of pages are uselessly written every year ; what careful prunings, what innumerable transcriptions and corrections and recorrections ; what calculations are made on the "acceptance," and what blank disappointment is a destined result in nine cases out of ten. It is authoritatively stated that within two years two thousand four hundred manuscripts were sent to a single magazine, fifteen hundred of which required less than five minutes each to decide upon their rejection. Five hundred more had to be read through before it could be decided that they were not worth printing, and of the whole only two hundred and eighty were used, about one in nine ; and perhaps not more than ten, out of those two hundred and eighty, were considered worth republication or being copied into other papers.

The lesson of the article is, "Don't expect much ;" or, as a Fifteenth Amendment expresses it, "Blessed am day what expects nuthin', caze they ain't aguine to be diserpinted."

